# The prognostic and predictive value of the luminal-like subtype in hormone receptor-positive breast cancer: an analysis of the DATA trial

**DOI:** 10.1016/j.esmoop.2025.104154

**Published:** 2025-02-07

**Authors:** S.W.M. Lammers, S.M.E. Geurts, K.E.P.E. Hermans, L.F.S. Kooreman, A.C.P. Swinkels, C.H. Smorenburg, M.J.C. van der Sangen, J.R. Kroep, A.H. Honkoop, F.W.P.J. van den Berkmortel, W.K. de Roos, S.C. Linn, A.L.T. Imholz, I.J.H. Vriens, V.C.G. Tjan-Heijnen, Dutch Breast Cancer Research Group (BOOG) for the DATA investigators

**Affiliations:** 1 Department of Medical Oncology, Maastricht University Medical Centre, GROW, Maastricht University, Maastricht, The Netherlands; 2 Department of Pathology, GROW School for Oncology and Reproduction, Maastricht University Medical Centre+, Maastricht, The Netherlands; 3 Clinical Research Department, Netherlands Comprehensive Cancer Organisation (IKNL), Nijmegen, The Netherlands; 4 Department of Medical Oncology, Netherlands Cancer Institute, Amsterdam, The Netherlands; 5 Department of Radiation Oncology, Catharina Hospital, Eindhoven, The Netherlands; 6 Department of Medical Oncology, Leiden University Medical Centre, Leiden, The Netherlands; 7 Department of Medical Oncology, Isala Clinics, Zwolle, The Netherlands; 8 Department of Medical Oncology, Zuyderland Medical Centre Heerlen-Sittard-Geleen, Geleen, The Netherlands; 9 Department of Surgery, Gelderse Vallei Hospital, Ede, The Netherlands; 10 Department of Pathology, University Medical Centre Utrecht, Utrecht, The Netherlands; 11 Department of Medical Oncology, Deventer Hospital, Deventer, The Netherlands

**Keywords:** breast neoplasms, aromatase inhibitors, luminal-like subtype, Ki-67 antigen, prognosis

## Abstract

**Background:**

This study determines the prognostic value of the luminal-like subtype in patients with hormone receptor-positive breast cancer and explores whether the efficacy of extended anastrozole therapy differs between patients with luminal A-like versus luminal B-like tumours.

**Materials and methods:**

The phase III DATA study (NCT00301457) examined the efficacy of 6 versus 3 years of anastrozole in postmenopausal women with early-stage hormone receptor-positive breast cancer who had received 2-3 years of tamoxifen. Patients with available formalin-fixed paraffin-embedded tissue blocks were identified and classified by immunohistochemical luminal-like subtype. Distant recurrence (DR) and breast cancer-specific mortality (BCSM) were compared by luminal-like subtype and treatment arm using competing risk methods.

**Results:**

This study included 788 patients: 491 had a luminal A-like tumour and 297 had a luminal B-like tumour. The median follow-up time was 13.1 years. Patients with luminal B-like tumours experienced a higher risk of DR [subdistribution hazard ratio (sHR) 1.44, 95% confidence interval (CI) 1.03-2.01, *P* = 0.03] and BCSM (sHR 1.68, 95% CI 1.15-2.45, *P* = 0.008) than patients with luminal A-like tumours. The efficacy of extended anastrozole therapy differed between patients with luminal A-like tumours (DR: sHR 0.51, 95% CI 0.30-0.88, *P* = 0.02; BCSM: sHR 0.39, 95% CI 0.19-0.82, *P* = 0.01) and patients with luminal B-like tumours (DR: sHR 2.09, 95% CI 0.96-4.53, *P* = 0.06; BCSM: sHR 2.36, 95% CI 0.80-7.00, *P* = 0.12) (*P*-interaction = 0.03 and *P*-interaction = 0.06, respectively).

**Conclusion:**

In patients with hormone receptor-positive breast cancer, the luminal B-like subtype was associated with a significantly worse prognosis when compared with the luminal A-like subtype. Extended anastrozole therapy halved the risk of DR and BCSM in patients with luminal A-like tumours, whereas no effect was seen in patients with luminal B-like tumours.

## Highlights

• Patients with luminal B-like tumours experience a significantly worse prognosis than patients with luminal A-like tumours.
• The prognostic impact of the luminal B-like subtype differed substantially between years 0-5 and years 5-15.
• Extended anastrozole therapy reduced BCSM by 60% in patients with luminal A-like tumours.
• Patients with luminal B-like tumours did not experience any benefit from extended anastrozole therapy.

## Introduction

Hormone receptor-positive breast cancer is associated with a lifelong risk of recurrence.^1^^,^^2^ Prognostic factors provide additional information about someone’s individual risk of recurrence. Traditional prognostic factors include tumour size, nodal status, and histological grade.^1^^,^^3^ Predictive factors provide information about someone’s expected benefit of systemic therapy. The estrogen receptor (ER) status and progesterone receptor (PR) status are important predictive factors for benefit of (extended) endocrine therapy, while the human epidermal growth factor receptor-2 (HER2) status is an important predictive factor for benefit of HER2-targeted therapy.^4^, ^5^, ^6^, ^7^, ^8^, ^9^, ^10^

The intrinsic molecular subtype may provide additional prognostic and predictive information. The majority of ER-positive (ER+) tumours are either classified as luminal A or luminal B.^11^, ^12^, ^13^, ^14^ Several studies have shown that disease outcomes of patients with luminal B tumours are inferior to those of patients with luminal A tumours.^11^, ^12^, ^13^^,^^15^, ^16^, ^17^ For example, in a *post hoc* analysis of the Women’s Healthy Eating and Living (WHEL) study, which classified 1253 breast cancer survivors according to PAM50 intrinsic molecular subtype, patients with luminal B tumours experienced a statistically significantly worse disease-free survival (DFS) [hazard ratio (HR) 1.60, 95% confidence interval (CI) 1.19-2.13] and breast cancer survival (HR 1.68, 95% CI 1.20-2.35) when compared with patients with luminal A tumours.^12^ Some studies, however, show that this adverse prognostic effect of the luminal B subtype is mainly observed during the first 5 years after diagnosis—a pattern which has also been observed for triple-negative breast cancer.^17^, ^18^, ^19^, ^20^ Furthermore, apart from being a prognostic factor, some studies suggest that the luminal subtype may be a predictive factor for long-term benefit of adjuvant treatment with tamoxifen.^18^^,^^21^ To the best of our knowledge, the predictive effect of the luminal subtype on the efficacy of (extended) aromatase inhibitors in the adjuvant setting has not been studied before.

The aim of the current exploratory analysis was to assess the prognostic and predictive value of the luminal-like subtype in the DATA study, a randomised controlled trial evaluating the efficacy of 6 versus 3 years of anastrozole in patients with hormone receptor-positive breast cancer who had received 2-3 years of tamoxifen.^8^^,^^22^ The first objective was to compare the outcomes of patients with luminal A-like tumours with those of patients with luminal B-like tumours during the total follow-up period (0-15 years), early follow-up period (0-5 years), and late follow-up period (5-15 years). The second objective was to explore whether the luminal-like subtype is a predictive factor for benefit of extended aromatase inhibition.

## Materials and methods

### Study population

The DATA study (NCT00301457) was a randomised controlled trial assessing the efficacy of 6 versus 3 years of anastrozole (1 mg orally once a day) in 1860 postmenopausal women with hormone receptor-positive breast cancer who were disease free after 2-3 years of tamoxifen.^8^^,^^22^ Eligible patients were recruited from 79 hospitals in the Netherlands between 2006 and 2009. At recruitment, trastuzumab was not yet considered the standard of care for patients with HER2-positive breast cancer. Details about the DATA study have been described previously.^8^^,^^22^

For the current study, all patients with available formalin-fixed paraffin-embedded (FFPE) tissue blocks were identified. Patients without sufficient material for assessment of the Ki-67 score and patients with ER-negative (ER−)/PR-positive (PR+) disease were excluded.

Follow-up assessments of the study population to monitor for disease recurrence or death were carried out twice yearly during the first 6 years after randomisation and once yearly thereafter. A mammogram was carried out every year. Database lock: 7 March 2022.

This study was carried out in line with the principles of the Declaration of Helsinki. Approval was granted by the medical ethics committee of the Radboud University Medical Centre, Nijmegen. Written informed consent was obtained from all patients.

### Pathology assessment

Tissue microarrays (TMAs) were created by extracting three 0.6 mm cores from all FFPE tissue blocks. One core was taken from the non-necrotic tumour centre, whereas two cores were taken from the pushing border. TMAs were subsequently cut into 0.5 μm slides and stained for the presence of the Ki-67 antigen using the MIB-1 antibody. The Ki-67 score of all three cores of the TMAs was graded by an experienced pathologist. In the current study, the highest value of all three Ki-67 scores was used for analysis.

### Definitions

Tumours were categorised by intrinsic subtype using immunohistochemical (IHC) measures of ER, PR, and Ki-67, and IHC and/or FISH measures of HER2. The ER and/or PR status were considered positive when ≥10% of cells had a positive nuclear staining of the ER and/or PR. The HER2 status was considered positive in case of an IHC score of 3+ or a positive FISH result. Luminal A-like disease was defined by ER positivity, PR positivity, HER2 negativity, and a low Ki-67 score (<14%), whereas luminal B-like disease was defined by ER positivity in combination with either PR negativity, HER2 positivity, or a high Ki-67 score (≥14%).^23^, ^24^, ^25^

### Endpoints

Distant recurrence (DR) and breast cancer-specific mortality (BCSM) were assessed as primary endpoints to minimise the impact of non-breast cancer-specific events on our research question. DFS and overall survival (OS) were assessed as secondary endpoints. DR was defined as time from randomisation to first occurrence of a DR. BCSM was defined as time from randomisation until death from breast cancer. DFS was defined as time from randomisation until breast cancer recurrence, second primary (breast) cancer, or death from any cause. OS was defined as time from randomisation until death from any cause.

### Statistical analysis

Baseline characteristics of the study population were compared by luminal-like subtype and treatment arm using the chi-square test.

The prognostic value of the luminal-like subtype was evaluated from randomisation onwards, whereas the predictive value of the luminal-like subtype on the efficacy of 6 versus 3 years of anastrozole was evaluated from 3 years after randomisation onwards (i.e. ‘adapted’ outcomes). Patients who were lost to follow-up or developed a DFS event during the first 3 years after randomisation were excluded from the predictive or ‘adapted’ analyses, as the adjuvant treatment did not differ in these first 3 years. The predictive analyses were carried out according to the intention-to-treat principle.

DR and BCSM were examined with the cumulative incidence function, Gray’s test, and multivariable Fine and Gray regression analyses, thereby considering non-breast cancer-related death as a competing event. DFS and OS were examined with the Kaplan–Meier method, log-rank test, and multivariable Cox regression analyses. Patients without an event were censored at the date of last follow-up in all analyses. The following confounding factors were included in the multivariable models: age, tumour status, nodal status, histology, and prior chemotherapy.

Prognostic analyses were carried out separately for the total follow-up period (years 0-15), early follow-up period (years 0-5), and late follow-up period (years 5-15).

The predictive effect of the luminal-like subtype on the association between extended anastrozole therapy and disease outcomes was evaluated by treatment-by-luminal-like-subtype interaction terms, calculated using likelihood ratio tests.

Additional sensitivity analyses which excluded patients with HER2-positive breast cancer were carried out for all endpoints to assess whether the inclusion of patients with HER2-positive breast cancer and the limited use of HER2-targeted therapy in this subgroup may have impacted the outcomes of patients with luminal B-like breast cancer.

All statistical analyses were carried out using SPSS (version 28), Stata (version 17), and RStudio (version 2023). *P* values were two-sided and considered statistically significant at a *P* value of ≤0.05.

## Results

The DATA study included 1860 eligible patients, of whom 884 patients had FFPE tissue blocks available for assessment of the Ki-67 score (Supplementary Figure S1, available at https://doi.org/10.1016/j.esmoop.2025.104154). Patients without sufficient material for assessment of the Ki-67 score were excluded (*n* = 84), as well as patients diagnosed with ER−/PR+ tumours (*n* = 12), leaving 788 patients eligible for the current study.

### Baseline characteristics

This study included 491 patients with luminal A-like tumours and 297 patients with luminal B-like tumours. Patients with luminal A-like tumours were less frequently diagnosed with node-negative disease (29.1% versus 39.7%; *P* = 0.002) and histological grade 3 tumours (21.8% versus 50.2%; *P* = <0.001) when compared with patients with luminal B-like tumours (Table 1). In the luminal B-like subgroup, 164 (55.2%) patients had a high Ki-67 score, 175 (58.9%) patients had ER+/PR− disease, and 27 (9.1%) patients had HER2-positive disease. Overall, two patients with HER2-positive breast cancer received HER2-targeted therapy. Baseline characteristics of the 788 patients included in the current study were similar to the baseline characteristics of the 1860 patients included in the DATA study (data not shown).

**Table 1:** Baseline characteristics of the study population according to luminal-like subtype [*n* (%)]

Characteristic	Luminal A-like (*n* = 491)	Luminal B-like (*n* = 297)	*P* value
Age at randomisation			0.86
<60 years	281 (57.2)	168 (56.6)	
≥60 years	210 (42.8)	129 (43.4)	
Tumour status			0.54
T1	238 (48.5)	132 (44.4)	
T2	216 (44.0)	142 (47.8)	
T3/4	37 (7.5)	23 (7.7)	
Nodal status			0.002
Negative	143 (29.1)	118 (39.7)	
Positive	348 (70.9)	179 (60.3)	
Histological grade			<0.001
Grade 1	113 (23.4)	26 (8.8)	
Grade 2	264 (54.8)	122 (41.1)	
Grade 3	105 (21.8)	149 (50.2)	
Ki-67 score			<0.001
<14%	491 (100.0)	133 (44.8)	
≥14%	0 (0.0)	164 (55.2)	
Hormone receptor status			<0.001
ER+/PR+	491 (100.0)	122 (41.1)	
ER+/PR-	0 (0.0)	175 (58.9)	
HER2 status			<0.001
Negative	491 (100.0)	270 (90.9)	
Positive	0 (0.0)	27 (9.1)	
Histology			0.08
Ductal	367 (74.7)	238 (80.1)	
Other	124 (25.3)	59 (19.9)	
Breast-conserving surgery			0.19
Yes	247 (50.3)	135 (45.5)	
No	244 (49.7)	162 (54.5)	
Prior chemotherapy			0.26
Yes	325 (66.2)	208 (70.0)	
No	166 (33.8)	89 (30.0)	
Treatment duration of anastrozole			0.37
3 years	245 (49.9)	158 (53.2)	
6 years	246 (50.1)	139 (46.8)	

Missing values: histological grade (*n* = 9).

ER, estrogen receptor; HER2, human epidermal growth factor receptor 2; PR, progesterone receptor.

### The prognostic value of the luminal-like subtype

The median follow-up time beyond randomisation was 13.1 years [interquartile range (IQR) 12.5-13.8 years], during which 150 patients developed a DR and 115 patients died of breast cancer. Detailed information about endpoint events is presented in Supplementary Table S1, available at https://doi.org/10.1016/j.esmoop.2025.104154.

**Figure 1:**
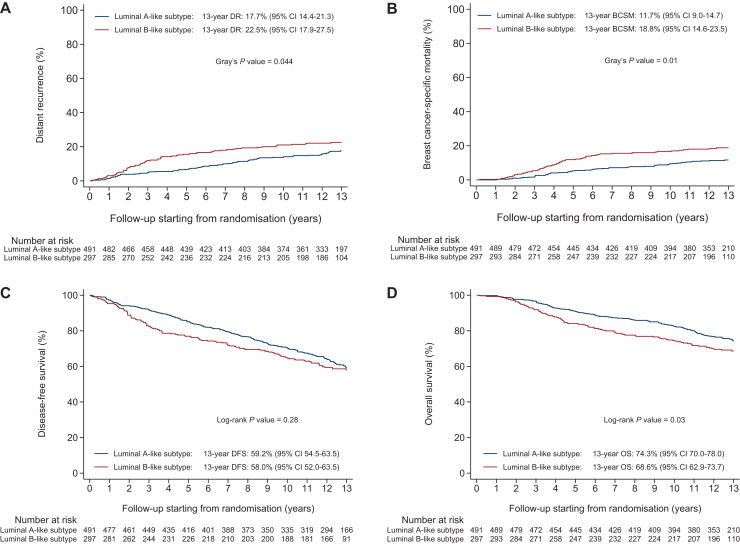
Distant recurrence (DR) (A), breast cancer-specific mortality (BCSM) (B), disease-free survival (DFS) (C), and overall survival (OS) (D) according to luminal-like subtype. CI, confidence interval.

The 13-year risk of DR was 17.7% (95% CI 14.4% to 21.3%) in patients with luminal A-like tumours and 22.5% (95% CI 17.9% to 27.5%) in patients with luminal B-like tumours (Figure 1A). Patients with luminal B-like tumours experienced a statistically significantly higher risk of DR [subdistribution hazard ratio (sHR) 1.44, 95% CI 1.03-2.01, *P* = 0.03] than patients with luminal A-like tumours, but the risk differed substantially between years 0-5 (sHR 1.45, 95% CI 0.90-2.35, *P* = 0.13) and years 5-15 (sHR 0.72, 95% CI 0.42-1.24, *P* = 0.23) (Table 2).

**Table 2:** Univariable and multivariable analyses assessing the prognostic association between luminal-like subtype and disease outcomes in the total study population

	Univariable analyses		Multivariable analyses^a^		Multivariable analyses^a^ (≤5 years)		Multivariable analyses^a^ (5-15 years)	
	(s)HR (95% CI)	*P* value	(s)HR (95% CI)	*P* value	(s)HR (95% CI)	*P* value	(s)HR (95% CI)	*P* value
Distant recurrence (*n* = 150 events)								
Luminal B-like versus luminal A-like subtype	1.39 (1.01-1.92)	0.05	1.44 (1.03-2.01)	0.03	1.45 (0.90-2.35)	0.13	0.72 (0.42-1.24)	0.23
Breast cancer-specific mortality (*n* = 115 events)								
Luminal B-like versus luminal A-like subtype	1.62 (1.12-2.33)	0.01	1.68 (1.15-2.45)	0.008	1.77 (1.03-3.05)	0.04	1.06 (0.58-1.91)	0.86
Disease-free survival (*n* = 323 events)								
Luminal B-like versus luminal A-like subtype	1.14 (0.91-1.43)	0.25	1.15 (0.92-1.44)	0.23	1.67 (1.19-2.33)	0.003	0.84 (0.61-1.16)	0.29
Overall survival (*n* = 221 events)								
Luminal B-like versus luminal A-like subtype	1.30 (0.99-1.69)	0.06	1.30 (0.99-1.70)	0.06	1.86 (1.23-2.82)	0.004	0.99 (0.69-1.43)	0.97

In the analyses of distant recurrence and breast cancer-specific mortality, we reported sHR instead of HR.

CI, confidence interval; (s)HR, (subdistribution) hazard ratio.

a Analyses were adjusted for age, tumour status, nodal status, histology, and prior chemotherapy.

Patients with luminal B-like tumours also experienced a statistically significantly higher risk of BCSM when compared with patients with luminal A-like tumours (sHR 1.68, 95% CI 1.15-2.45, *P* = 0.008), but again results differed between years 0-5 (sHR 1.77, 95% CI 1.03-3.05, *P* = 0.04) and years 5-15 (sHR 1.06, 95% CI 0.58-1.91, *P* = 0.86) (Figure 1B and Table 2).

The luminal B- versus A-like subtype was not associated with DFS (HR 1.15, 95% CI 0.92-1.44, *P* = 0.23) during the total follow-up period (Figure 1C and Table 2). We, however, observed that patients with luminal B-like tumours experienced a statistically significant decrease in DFS (HR 1.67, 95% CI 1.19-2.33, *P* = 0.003) during years 0-5, whereas no effect on DFS (HR 0.84, 95% CI 0.61-1.16, *P* = 0.29) was observed during years 5-15 (Table 2).

The luminal B- versus A-like subtype was associated with a trend towards a worse OS (HR 1.30, 95% CI 0.99-1.70, *P* = 0.06) during the total follow-up period (Figure 1D and Table 2). In line with the results of other endpoints, the association between the luminal B-like subtype and OS differed between years 0-5 (HR 1.86, 95% CI 1.23-2.82, *P* = 0.004) and years 5-15 (HR 0.99, 95% CI 0.69-1.43, *P* = 0.97) (Table 2).

In additional sensitivity analyses which excluded patients with HER2-positive breast cancer, similar associations between the luminal-like subtype and all endpoints were observed in both the total follow-up period and specific follow-up periods (years 0-5 versus years 5-15) (Supplementary Table S2, available at https://doi.org/10.1016/j.esmoop.2025.104154).

### The predictive value of the luminal-like subtype

Baseline characteristics of the study population according to assigned treatment are presented separately for patients with luminal A-like tumours and patients with luminal B-like tumours (Supplementary Tables S3 and S4, available at https://doi.org/10.1016/j.esmoop.2025.104154). In patients with luminal A-like tumours, baseline characteristics were similar between treatment arms, except for age: 63.9% of patients in the 6-year arm versus 53.2% of patients in the 3-year arm were younger than 60 years of age (*P* = 0.02) (Supplementary Table S3, available at https://doi.org/10.1016/j.esmoop.2025.104154). In patients with luminal B-like tumours, age also differed between treatment arms: 48.2% of patients in the 6-year arm versus 62.1% of patients in the 3-year arm were younger than 60 years of age (*P* = 0.03) (Supplementary Table S4, available at https://doi.org/10.1016/j.esmoop.2025.104154).

**Figure 2:**
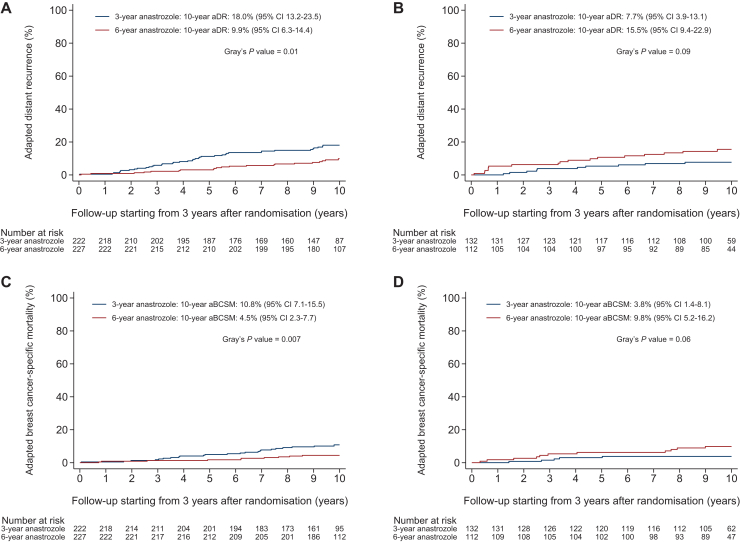
Adapted distant recurrence (aDR) in patients with luminal A-like tumours (A) and patients with luminal B-like tumours (B) and adapted breast cancer-specific mortality (aBCSM) in patients with luminal A-like tumours (C) and patients with luminal B-like tumours (D) according to assigned treatment from 3 years after randomisation onwards. CI, confidence interval.

**Figure 3:**
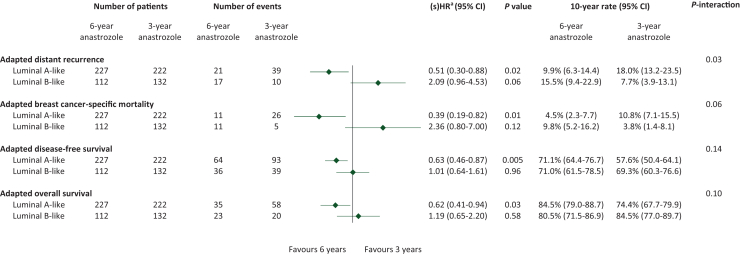
Multivariable analyses of adapted distant recurrence, adapted breast cancer-specific mortality, adapted disease-free survival, and adapted overall survival evaluating the efficacy of 6 versus 3 years of anastrozole from 3 years after randomisation onwards, stratified by luminal-like subtype. CI, confidence interval; s(HR), subdistribution hazard ratio.^a^Analyses were adjusted for age, tumour status, nodal status, histology, and prior chemotherapy.

The efficacy of extended anastrozole therapy differed between patients with luminal A-like tumours and patients with luminal B-like tumours. In patients with luminal A-like tumours, the 10-year adapted risk of DR was statistically significantly lower in patients who received 6 [9.9% (95% CI 6.3% to 14.4%)] versus 3 years of anastrozole [18.0% (95% CI 13.2% to 23.5%)] (adjusted sHR 0.51, 95% CI 0.30-0.88, *P* = 0.02) (Figures 2A and 3). By contrast, in patients with luminal B-like tumours, the adapted risk of DR was numerically higher in patients who received 6 versus 3 years of anastrozole (adjusted sHR 2.09, 95% CI 0.96-4.53, *P* = 0.06) (Figures 2B and 3). The treatment-by-luminal-like subtype interaction term for adapted DR was statistically significant (*P* = 0.03) (Figure 3).

Similarly, in patients with luminal A-like tumours, the 10-year adapted risk of BCSM was statistically significantly lower in patients who received 6 [4.5% (95% CI 2.3% to 7.7%)] versus 3 years of anastrozole [10.8% (95% CI 7.1% to 15.5%)] (adjusted sHR 0.39, 95% CI 0.19-0.82, *P* = 0.01) (Figures 2C and 3). In patients with luminal B-like tumours, however, the risk of BCSM was numerically higher in patients who received 6 versus 3 years of anastrozole (adjusted sHR 2.36, 95% CI 0.80-7.00, *P* = 0.12) (Figures 2D and 3). The *P*-interaction term for adapted BCSM was 0.06 (Figure 3).

In the analyses of adapted DFS and adapted OS, the efficacy of 6 versus 3 years of anastrozole also differed between patients with luminal A-like tumours and patients with luminal B-like tumours, but *P*-interaction terms were not statistically significant (Figure 3, Supplementary Figure S2A-D, available at https://doi.org/10.1016/j.esmoop.2025.104154).

Findings remained similar after excluding patients with HER2-positive breast cancer (Supplementary Figure S3, available at https://doi.org/10.1016/j.esmoop.2025.104154).

## Discussion

In this exploratory analysis including 788 patients with hormone receptor-positive breast cancer from the phase III DATA study on the extended use of anastrozole, we revealed that the luminal B-like subtype is an important adverse prognostic factor for DR and BCSM. The prognostic impact differed substantially between years 0-5 and years 5-15 beyond randomisation. Even more important, our results suggest that the luminal-like subtype can be used as a predictive factor for benefit of extended anastrozole therapy. We observed that patients with luminal A-like tumours experienced a large benefit from extended anastrozole therapy, in contrast to patients with luminal B-like tumours, who did not seem to derive any benefit at all.

Over the years, several studies evaluated the prognostic potential of the IHC-based intrinsic subtype.^15^^,^^17^^,^^25^, ^26^, ^27^ A retrospective observational cohort study by Cheang et al.^25^ including 943 patients with node-negative, hormone receptor-positive breast cancer who did not receive systemic therapy, for example, showed that patients with luminal B-like/HER2-negative and luminal B-like/HER2-positive tumours experience a statistically significant deterioration in relapse-free survival (HR 1.40, 95% CI 1.10-1.90 and HR 1.60, 95% CI 1.00-2.50) and breast cancer-specific survival (HR 1.80, 95% CI 1.30-2.60 and HR 2.10, 95% CI 1.20-3.80) when compared with patients with luminal A-like tumours.^25^ In the current study, we also observed that patients with luminal B-like tumours experienced a higher risk of DR (sHR 1.44, 95% CI 1.03-2.01) and BCSM (sHR 1.68, 95% CI 1.15-2.45) when compared with patients with luminal A-like tumours. Interestingly, we only observed an increased risk of DR and BCSM in patients with luminal B-like versus luminal A-like tumours during the first 5 years after randomisation, but not thereafter. Few other studies have evaluated whether the recurrence risk of luminal B versus A tumours differs by time of follow-up.^14^^,^^17^^,^^18^ In a study among 786 patients with tamoxifen-treated ER+ breast cancer, breast cancer-specific survival was worse for patients with luminal B versus A tumours, but the increased risk was present both during the early and later years of follow-up.^14^ In a case-cohort study of 1638 patients with breast cancer, however, patients with luminal B tumours experienced a higher early versus late recurrence risk (HR 2.55, 95% CI 1.68-3.88 versus HR 1.34, 95% CI 0.79-2.28) and a higher early versus late BCSM risk (HR 2.67, 95% CI 1.46-4.88 versus HR 1.47, 95% CI 0.87-2.48) when compared with patients with luminal A tumours.^17^ Correspondingly, a secondary analysis of the Stockholm tamoxifen trial showed that the risk of recurrence of patients with untreated luminal B tumours was considerably higher during the first 5 years after diagnosis than in the later years.^18^ The findings of these studies and those of our study suggest that the luminal B-like subtype behaves more like the HER2-enriched or basal-like subtype, where recurrences are mainly observed during the first years after diagnosis.^14^^,^^17^^,^^19^^,^^28^ The disease behaviour of luminal B-like tumours, and especially the risk of early recurrence, should be considered in patient counselling and demands the use of effective systemic therapies at the time of diagnosis.

Results of our study furthermore suggest that the luminal-like subtype is a predictive factor for benefit of extended anastrozole therapy. We observed that patients with luminal A-like tumours experienced a large benefit of extended anastrozole therapy (sHR for DR 0.51, 95% CI 0.30-0.88), whereas patients with luminal B-like tumours did not seem to derive any benefit from extended anastrozole therapy (sHR for DR 2.09, 95% CI 0.96-4.53). A recent retrospective observational cohort study, using data from the Stockholm tamoxifen trial 3, has evaluated the association between PAM50-based luminal subtype and long-term benefit of 2-5 years of adjuvant tamoxifen.^18^ In that study, it was shown that patients with luminal A and B tumours derived a similar distant recurrence-free survival (DRFS) benefit from tamoxifen during the first 5 years after diagnosis.^18^ However, the 15-year DRFS benefit of tamoxifen differed substantially between patients with luminal A tumours (HR 0.57, 95% CI 0.35-0.94) and patients with luminal B tumours (HR 1.04, 95% CI 0.38-2.82).^18^ Some evidence for a comparable differential effect on breast cancer outcome for aromatase inhibitor therapy can be derived from subgroup analyses of five randomised controlled trials, in which the PR status and/or MammaPrint score were considered.^7^^,^^8^^,^^29^, ^30^, ^31^ In the MA.17 trial, GIM-4 trial, and DATA trial, for example, the benefit of extended aromatase inhibition seemed to be largely limited to patients with ER+/PR+ tumours.^7^^,^^8^^,^^29^ The NSABP B-42 trial and IDEAL trial furthermore showed that extended aromatase inhibition was only beneficial in patients with a low-risk MammaPrint score.^30^^,^^31^ As luminal B tumours are to a large extent characterised by a negative PR status and a high-risk MammaPrint score, these findings support our present results on the lack of efficacy of extended aromatase inhibition in patients with luminal B-like tumours.

Differences in the efficacy of endocrine therapy may be explained by differences in the biology of luminal A versus B tumours.^6^^,^^32^, ^33^, ^34^ The main tumour characteristics that distinguish luminal B tumours from luminal A tumours include a higher level of proliferation, PR negativity, and/or HER2 positivity.^35^^,^^36^ One mechanism of endocrine resistance in luminal B tumours may be related to a cross-talk between tyrosine kinase receptors and the ER and/or PR.^6^^,^^37^ In this cross-talk theory, upstream tyrosine kinase receptor activation, for instance by amplification of *EGFR*, *HER2*, *IGFR1*, or *FGFR1*, results in activation of the *PI3K-Akt-mTOR* pathway, *p42/44 MAPK* pathway, and several other proliferation pathways.^6^^,^^37^ These pathways subsequently result in a reduced expression of the PR (i.e. PR-negative disease by IHC testing), up-regulation of proliferation factors (i.e. a high Ki-67 value or a high genomic risk score), and ultimately endocrine resistance following ligand-independent activation of the ER.^6^^,^^37^ *TP53* mutations, *ATM* loss, *MDM2* amplification, and *cyclin D1* amplification are also shown to occur more frequently in luminal B versus A tumours.^32^ In addition, estrogen receptor 1 (*ESR1*) mutations may be important in unravelling differences in the efficacy of (extended) aromatase inhibition in patients with luminal A versus B tumours. In the metastatic setting, the presence of *ESR1* mutations is associated with resistance to aromatase inhibitors.^38^^,^^39^ Interestingly, recent studies showed that *ESR1* mutations occur more frequently in luminal B versus A tumours.^34^^,^^40^ We hypothesise that extended aromatase inhibition may further increase the likelihood of developing *ESR1* mutations in luminal B tumours. Escalation of initial systemic therapy, for example by adding a cyclin-dependent kinase (CDK) 4/6 inhibitor, may therefore be a more appropriate treatment option for patients with luminal B tumours.^41^^,^^42^ It is important to note that CDK 4/6 inhibitors have shown to be equally effective in luminal A and B tumours.^43^, ^44^, ^45^, ^46^, ^47^

Strengths of our study include the randomised controlled study design and long-term follow-up period of currently >13 years beyond randomisation. Our study also has certain limitations. We, for example, did not have FFPE tissue blocks available from all patients included in the DATA study and used TMAs instead of whole tissue sections for assessment of the Ki-67 score. Studies have, however, shown that TMAs can safely be used for assessment of the Ki-67 score.^48^^,^^49^ The use of an IHC-based intrinsic subtype classification instead of a PAM50-based intrinsic molecular subtype classification can be considered another limitation of our study. None the less, an IHC-based classification can easily be used in daily clinical practice, whereas the use of gene expression assays is limited by high costs and restricted access in developing countries. In addition, our study population was too small to carry out any subgroup analyses within the luminal B-like subgroup. Obviously, the luminal B-like subgroup comprised a wide variety of tumours which likely have a differential prognosis when analysed separately. Finally, the start of follow-up, i.e. 2-3 years beyond diagnosis of breast cancer, may have introduced selection bias as patients who developed an early DFS event were excluded from our study. We expect that the adverse prognostic effect of the luminal B- versus A-like subtype might even have been stronger when patients were included from diagnosis onwards.

To conclude, in this exploratory analysis among 788 postmenopausal women with hormone receptor-positive breast cancer from the phase III DATA study on the extended use of anastrozole, we observed that patients with luminal B-like tumours experience a higher risk of DR and BCSM when compared with patients with luminal A-like tumours, and that this risk is highest during the first years after diagnosis. In addition, we observed that extended anastrozole therapy was highly effective in patients with luminal A-like tumours, where the risk of DR and BCSM was halved, in contrast to patients with luminal B-like tumours, who did not derive any benefit. Taking the evidence altogether, we consider extended use of endocrine therapy including an aromatase inhibitor as a good treatment strategy for patients with luminal A-like breast cancer, whereas additional targeted therapies may be indicated for patients with luminal B-like tumours.
