# Supplementary material for: The prognostic and predictive value of the luminal-like subtype in hormone receptor-positive breast cancer: an analysis of the DATA trial
Source: ESMO Open. 2025 Feb 7;10(2):104154. doi: 10.1016/j.esmoop.2025.104154 (PMC11850755; doi:10.1016/j.esmoop.2025.104154)
Supplement: Supplementary Material [file mmc1.pdf]

Supplementary appendix

Supplement to: Lammers S.W.M., Geurts S.M.E., Hermans K.E.P.E., et al. The prognostic and predictive value of the luminal-like subtype in hormone receptor-positive breast cancer: an analysis of the DATA trial.

Corresponding author:
Vivianne C.G. Tjan-Heijnen, MD, PhD
ORCiD ID: 0000-0002-3935-7440
Department of Medical Oncology, Maastricht University Medical Centre
P.O. Box 5800, 6202 AZ Maastricht, the Netherlands
Email: vcg.tjan.heijnen@mumc.nl
Phone: +31 433877025

Table of contents

Supplementary Figure 1. Flowchart of included patients 3

Supplementary Table 1. Endpoint events in the study population 4

Supplementary Table 2. Univariable and multivariable analyses assessing the prognostic association between luminal-like subtype and disease outcomes in patients with HR+/HER2-negative breast cancer. 5

Supplementary Table 3. Baseline characteristics of patients with luminal A-like tumours who were disease-free at three years after randomisation according to assigned treatment (N (%)) 6

Supplementary Table 4. Baseline characteristics of patients with luminal B-like tumours who were disease-free at three years after randomisation according to assigned treatment (N (%)) 7

Supplementary Figure 2. Adapted disease-free survival in patients with luminal A-like tumours (A) and patients with luminal B-like tumours (B) and adapted overall survival in patients with luminal A-like tumours (C) and patients with luminal B-like tumours (D), according to assigned treatment from three years after randomisation onwards 8

Supplementary Figure 3. Multivariable analyses of adapted distant recurrence, adapted breast cancer-specific mortality, adapted disease-free survival, and adapted overall survival evaluating the efficacy of six versus three years of anastrozole in patients with HR+/HER2-negative breast cancer from three years after randomisation onwards, stratified by luminal-like subtype. 9

Supplementary Figure 1. Flowchart of included patients

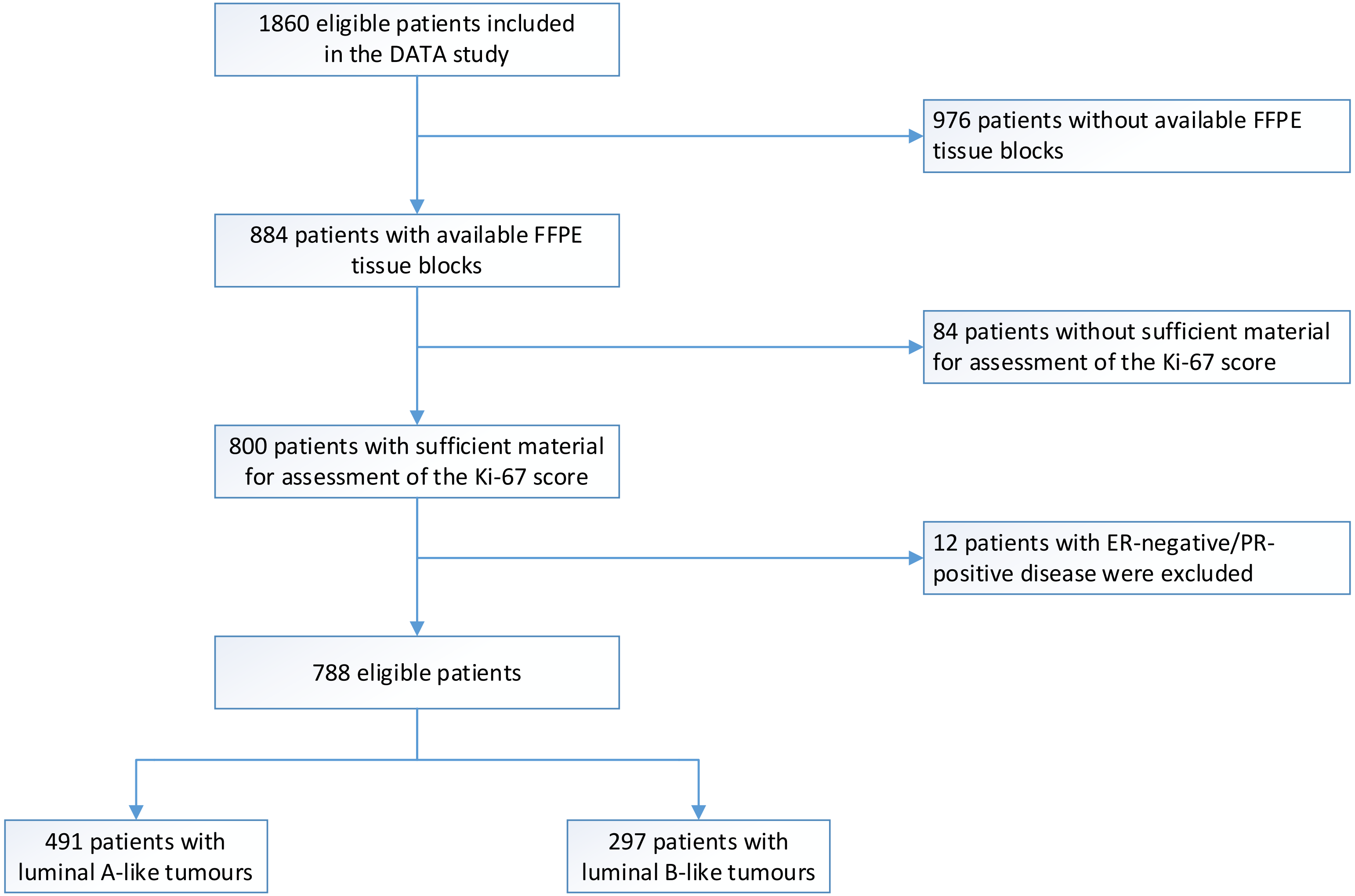

Abbreviations: ER = oestrogen receptor; FFPE = formalin-fixed paraffin embedded, PR = progesterone receptor

Supplementary Table 1. Endpoint events in the study population

	Luminal A-like (N=491)	Luminal B-like (N=297)
**Disease-free survival event[1]**	197	126
Recurrence of the primary tumour	89 (45.2)	71 (56.3)
Local recurrence	10 (5.1)	14 (11.1)
Regional recurrence	17 (8.6)	19 (15.1)
Distant recurrence[2]	75 (38.1)	55 (43.7)
Visceral	47 (23.9)	27 (21.4)
Bone	45 (22.8)	35 (27.8)
Soft tissue	16 (8.1)	7 (5.6)
Other	2 (1.0)	1 (0.8)
Second primary breast cancer	18 (9.1)	11 (8.7)
Second primary cancer	59 (29.9)	28 (22.2)
Death without prior breast cancer event	36 (18.3)	17 (13.5)
**Distant recurrence[3]**	84	66
**Death from any cause**	128	93
Breast cancer-related death	60 (46.9)	55 (59.1)
Non-breast cancer-related death	68 (53.1)	38 (40.9)
Second primary malignancy	25 (19.5)	19 (20.4)
Cardiovascular disease	9 (7.0)	7 (7.5)
Other	34 (26.6)	12 (12.9)

[1]Multiple disease-free survival events may have occurred at the same moment.
[2]In some patients multiple locations of distant recurrence were reported.
[3]Distant recurrence events at any time during follow-up, including events which occurred after a prior locoregional recurrence or second primary (breast) cancer.

Supplementary Table 2. Univariable and multivariable analyses assessing the prognostic association between luminal-like subtype and disease outcomes in patients with HR+/HER2-negative breast cancer.

	Univariable analyses		Multivariable analyses[1]		Multivariable analyses[1] (≤5 years)		Multivariable analyses[1] (5+ years)	
	**(s)HR (95% CI)**	**P-value**	**(s)HR (95% CI)**	**P-value**	**(s)HR (95% CI)**	**P-value**	**(s)HR (95% CI)**	**P-value**
**Distant recurrence (n = 145 events)**								
Luminal B-like versus luminal A-like subtype	1.42 (1.02-1.98)	0.04	1.43 (1.01-2.01)	0.04	1.49 (0.92-2.42)	0.10	0.73 (0.42-1.28)	0.27
**Breast cancer-specific mortality (n = 111 events)**								
Luminal B-like versus luminal A-like subtype	1.66 (1.14-2.41)	0.008	1.66 (1.13-2.45)	0.01	1.80 (1.05-3.09)	0.03	1.01 (0.54-1.88)	0.98
**Disease-free survival (n = 312 events)**								
Luminal B-like versus luminal A-like subtype	1.15 (0.92-1.45)	0.22	1.14 (0.90-1.44)	0.27	1.64 (1.16-2.31)	0.005	0.84 (0.61-1.17)	0.30
**Overall survival (n = 212 events)**								
Luminal B-like versus luminal A-like subtype	1.29 (0.98-1.71)	0.07	1.26 (0.95-1.67)	0.10	1.84 (1.20-2.81)	0.005	0.95 (0.65-1.38)	0.77

Abbreviations: CI = confidence interval; (s)HR = (subdistribution) hazard ratio.

In the analyses of distant recurrence and breast cancer-specific mortality, we reported sHR instead of HR.
[1]Analyses were adjusted for age, tumour status, nodal status, histology, and prior chemotherapy.

Supplementary Table 3. Baseline characteristics of patients with luminal A-like tumours who were disease-free at three years after randomisation according to assigned treatment (N (%))

Characteristic		6-year anastrozole (N=227)	3-year anastrozole (N=222)	P-value
Age at randomisation				0.02
	<60 years	145 (63.9)	118 (53.2)	
	≥60 years	82 (36.1)	104 (46.8)	
Tumour status				0.43
	T1	109 (48.0)	109 (49.1)	
	T2	105 (46.3)	94 (42.3)	
	T3/4	13 (5.7)	19 (8.6)	
Nodal status				0.88
	Negative	65 (28.6)	65 (29.3)	
	Positive	162 (71.4)	157 (70.7)	
Histological grade				0.54
	Grade 1	56 (25.1)	53 (24.4)	
	Grade 2	127 (57.0)	116 (53.5)	
	Grade 3	40 (17.9)	48 (22.1)	
Ki-67 score				-
	<14%	227 (100.0)	222 (100.0)	
	≥14%	0 (0.0)	0 (0.0)	
Hormone receptor status				-
	ER+/PR+	227 (100.0)	222 (100.0)	
	ER+/PR-	0 (0.0)	0 (0.0)	
HER2 status				-
	Negative	227 (100.0)	222 (100.0)	
	Positive	0 (0.0)	0 (0.0)	
Histology				0.89
	Ductal	170 (74.9)	165 (74.3)	
	Other	57 (25.1)	57 (25.7)	
Breast-conserving surgery				0.43
	Yes	120 (52.9)	109 (49.1)	
	No	107 (47.1)	113 (50.9)	
Prior chemotherapy				0.24
	Yes	158 (69.6)	143 (64.4)	
	No	69 (30.4)	79 (35.6)	
Abbreviations: ER = oestrogen receptor, HER2 = human epidermal growth factor receptor 2, PR = progesterone receptor.				
Missing values: histological grade (n = 9).				

Supplementary Table 4. Baseline characteristics of patients with luminal B-like tumours who were disease-free at three years after randomisation according to assigned treatment (N (%))

Characteristic	6-year anastrozole (N=112)	3-year anastrozole (N=132)	P-value
Age at randomisation			0.03
<60 years	54 (48.2)	82 (62.1)	
≥60 years	58 (51.8)	50 (37.9)	
Tumour status			0.49
T1	57 (50.9)	57 (43.2)	
T2	47 (42.0)	64 (48.5)	
T3/4	8 (7.1)	11 (8.3)	
Nodal status			0.26
Negative	42 (37.5)	59 (44.7)	
Positive	70 (62.5)	73 (55.3)	
Histological grade			0.62
Grade 1	9 (8.0)	14 (10.6)	
Grade 2	44 (39.3)	56 (42.4)	
Grade 3	59 (52.7)	62 (47.0)	
Ki-67 score			0.08
<14%	60 (53.6)	56 (42.4)	
≥14%	52 (46.4)	76 (57.6)	
Hormone receptor status			0.34
ER+/PR+	40 (35.7)	55 (41.7)	
ER+/PR-	72 (64.3)	77 (58.3)	
HER2 status			0.62
Negative	103 (92.0)	119 (90.2)	
Positive	9 (8.0)	13 (9.8)	
Histology			0.85
Ductal	88 (78.6)	105 (79.5)	
Other	24 (21.4)	27 (20.5)	
Breast-conserving surgery			0.06
Yes	61 (54.5)	56 (42.4)	
No	51 (45.5)	76 (57.6)	
Prior chemotherapy			0.50
Yes	77 (68.8)	96 (72.7)	
No	35 (31.3)	36 (27.3)	

Abbreviations: ER = oestrogen receptor, HER2 = human epidermal growth factor receptor 2, PR = progesterone receptor.

Supplementary Figure 2. Adapted disease-free survival in patients with luminal A-like tumours (A) and patients with luminal B-like tumours (B) and adapted overall survival in patients with luminal A-like tumours (C) and patients with luminal B-like tumours (D), according to assigned treatment from three years after randomisation onwards

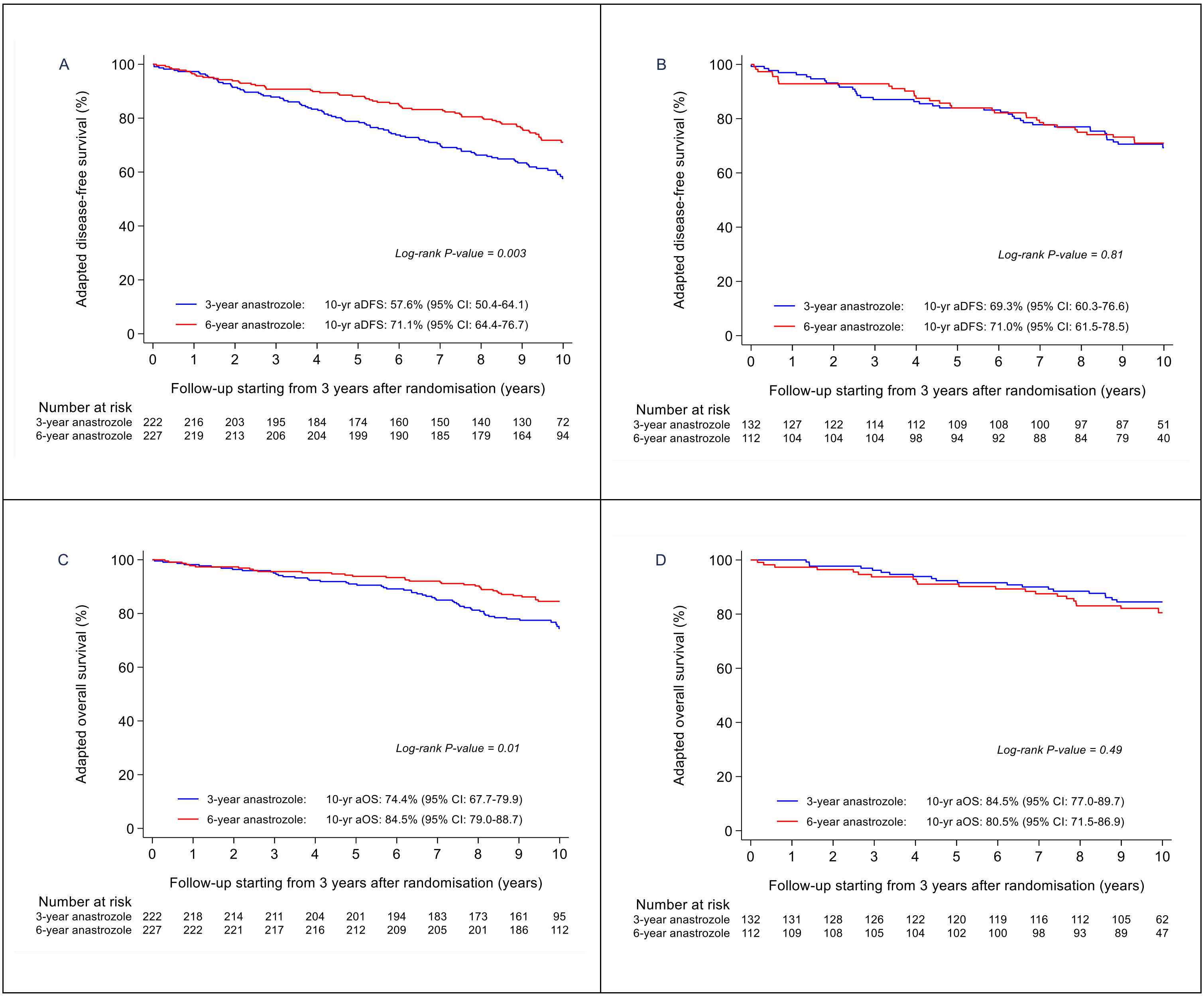

Supplementary Figure 3. Multivariable analyses of adapted distant recurrence, adapted breast cancer-specific mortality, adapted disease-free survival, and adapted overall survival evaluating the efficacy of six versus three years of anastrozole in patients with HR+/HER2-negative breast cancer from three years after randomisation onwards, stratified by luminal-like subtype.

	Number of patients		Number of events		(s)HR* (95% CI)	P-value	10-year rate (95% CI)		P interaction
	6-year anastrozole	3-year anastrozole	6-year anastrozole	3-year anastrozole			6-year anastrozole	3-year anastrozole	
**Adapted distant recurrence**									0.03
Luminal A-like	227	222	21	39	0.51 (0.30-0.88)	0.02	9.9% (6.3-14.4)	18.0% (13.2-23.5)	
Luminal B-like	103	119	16	9	2.04 (0.91-4.62)	0.09	16.0% (9.5-23.8)	7.7% (3.8-13.4)	
**Adapted breast cancer-specific mortality**									0.07
Luminal A-like	227	222	11	26	0.39 (0.19-0.82)	0.01	4.5% (2.3-7.7)	10.8% (7.1-15.5)	
Luminal B-like	103	119	10	5	2.01 (0.68-5.95)	0.21	9.7% (5.0-16.4)	4.2% (1.6-9.0)	
**Adapted disease-free survival**									0.18
Luminal A-like	227	222	64	93	0.63 (0.46-0.87)	0.005	71.1% (64.4-76.7)	57.6% (50.4-64.1)	
Luminal B-like	103	119	33	36	0.98 (0.61-1.59)	0.95	70.3% (60.3-78.3)	68.4% (58.8-76.2)	
**Adapted overall survival**									0.11
Luminal A-like	227	222	35	58	0.62 (0.41-0.94)	0.03	84.5% (79.0-88.7)	74.4% (67.7-79.9)	
Luminal B-like	103	119	21	18	1.17 (0.62-2.23)	0.63	79.6% (69.9-86.5)	84.5% (76.5-89.9)	

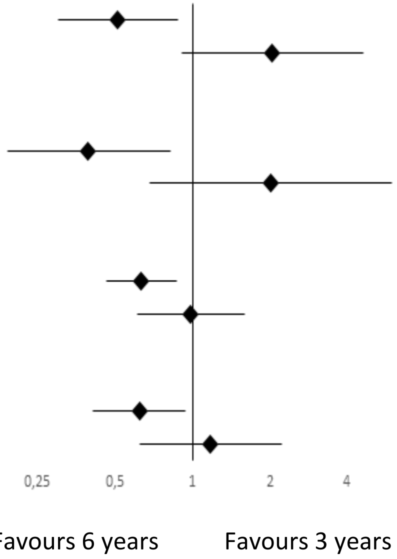

Abbreviations: CI = confidence interval; (s)HR = (subdistribution) hazard ratio.

*Analyses were adjusted for age, tumour status, nodal status, histology, and prior chemotherapy.
